# Transcriptomic meta-analysis identifies dysregulated pathways and potential therapeutic targets in Vestibular Schwannoma

**DOI:** 10.1371/journal.pone.0353343

**Published:** 2026-07-10

**Authors:** Ebrar Altınalan, Aleksandra Panina, Robert Fredriksson, Ayse Arzu Şakul, Helgi B. Schiöth

**Affiliations:** 1 Department of Medical Pharmacology, School of Medicine, Istanbul Medipol University, Istanbul, Turkiye; 2 Department of Pharmaceutical Biosciences, Uppsala University, Uppsala, Sweden; 3 Department of Medical Pharmacology, Istanbul Medipol University; Research Institute for Health Sciences and Technologies (SABITA), Istanbul, Turkiye; 4 Department of Surgical Sciences, Functional Pharmacology and Neuroscience, Uppsala University, Uppsala, Sweden; 5 Laboratory of Pharmaceutical Pharmacology, Latvian Institute of Organic Synthesis, Riga, Latvia; LSU Health Shreveport, UNITED STATES OF AMERICA

## Abstract

Vestibular schwannoma (VS) is a benign Schwann cell–derived tumor that frequently causes progressive hearing loss and vestibulocochlear dysfunction, substantially impacting quality of life. The molecular mechanisms underlying VS pathobiology remain poorly defined, and reliable biomarkers or targeted therapies are lacking. This study aimed to delineate the molecular landscape of VS through a transcriptome-wide meta-analysis. We performed a genome-wide random-effects meta-analysis of four independent Affymetrix microarray datasets from the Gene Expression Omnibus (GEO) database. Differential expression analyses were conducted with and without covariate adjustment. Gene Ontology enrichment and DrugBank-based drug–gene interaction analyses were subsequently applied to characterize biological pathways and assess translational potential. Across the meta-analysis, more than 3,200 differentially expressed genes were identified in the covariate-free model. After applying a more stringent threshold (|metaLFC| > 1 and FDR < 0.05), 1,095 genes remained differentially expressed, with high concordance between the covariate-free and covariate-adjusted models. Downregulated genes included extracellular matrix and stromal components (*MFAP5*, *FABP4*, *DCN*), and sensory- and synapse-related transcripts (*SLC22A3*, *LGI1*). Upregulated genes included immune- and inflammation-associated genes (*TREM2*, *CCL3*, *CCL4*, *L1CAM*) and proliferative regulators (*CCND1*, *RAB31*, *MOXD1*). Functional enrichment highlighted extracellular matrix remodeling, immune modulation, sensory signaling, and cell cycle pathways. Notably, many of the most strongly dysregulated genes have not previously been associated with VS. Drug–gene interaction analysis identified multiple dysregulated genes with known pharmacological targets, suggesting potential translational relevance. This transcriptome-wide meta-analysis provides a comprehensive overview of gene expression patterns in VS, highlighting alterations related to extracellular matrix organization, sensory and synaptic processes, immune-associated signaling, and cell cycle–related pathways. The study highlights novel disease-associated genes and pathways and may help prioritize candidates for further investigation, including those with potential relevance for therapeutic targeting.

## Introduction

Vestibular schwannoma (VS), also known as acoustic neuroma, is a type of tumor originating from the Schwann cells of the 8th cranial nerve [1]. Accounting for approximately 85% of cerebellopontine angle tumors, VS is a slow-growing benign neoplasm that nonetheless poses risks to adjacent intracranial structures due to its location [1]. The most common symptom is progressive hearing loss (~60%), with possible trigeminal neuralgia, vertigo, and headache due to brainstem compression [1,2]. There are two types of VS, unilateral sporadic and bilateral hereditary forms, and approximately 95% of these are sporadic and unilateral tumors [3].

VS is among the most prevalent benign intracranial tumors, following meningiomas and pituitary adenomas, with an estimated incidence of approximately 3.0–5.2 cases per 100 000 person‑years and a lifetime prevalence exceeding 1 in 500 individuals, reflecting increased detection and clinical recognition in recent years [4,5]. The primary molecular driver is biallelic NF2 inactivation, encoding the cytoskeletal regulator merlin; yet VS shows low mutational burden beyond NF2, with additional genetic and epigenetic contributors increasingly implicated [6]. Recent multi-omic studies reveal molecular heterogeneity, including immunogenic and proliferative subtypes, yet these studies have been limited by sample numbers and inconsistent replication across cohorts [7]. Single-cell transcriptomic profiling has uncovered diverse Schwann cell states—including injury-like and vascular endothelial growth factor A (VEGFA)-expressing subpopulations—associated with growth patterns and microenvironmental interactions, providing novel insight yet underscoring the complexity and incomplete understanding of VS pathogenesis [8,9].

A variety of therapeutic strategies are currently available for VS management. Wait-and-scan with periodic imaging is often the initial approach, while additional treatment options include pharmacological agents such as bevacizumab, everolimus, and lapatinib, as well as radiotherapy or surgery [1]. Although promising results have been reported on the use of bevacizumab in treating schwannoma, these data are based solely on cases of neurofibromatosis type 2 (NF2) [10]. Despite ongoing advances that have reduced morbidity and mortality in recent years, there remains no safe and effective pharmacological therapy capable of reliably improving tumor size or preserving hearing function [11]. Surgery is a good option for achieving tumor control in VS patients; however, it is among the most technically challenging tumors to remove from the cranial base [12]. On the other hand, the radiotherapy option carries risks such as radiation-induced tumor formation [13].

There are some previous studies that have investigated the transcriptomic profile of VS but these studies have largely been conducted with limited sample sizes based on single cohorts or have focused on specific biological aspects [14–16]. In a recent single-cell multi-omic study, the cellular heterogeneity of the VS microenvironment and the presence of “injury-like” Schwann cell states were characterized in detail [9]. Additionally, Sung and colleagues conducted a comprehensive study integrating bulk transcriptome data with single-cell RNA-seq analyses to elucidate tumor growth dynamics in VS and identified a 23-gene signature associated with growth rates [16]. While these studies offer important mechanistic and cell-type–specific perspectives, they remain focused on specific phenotypic aspects and do not address genome-wide transcriptional alterations across multiple independent cohorts. Also, an earlier integrated analysis of VS transcriptomic data explored both mRNA and microRNA expression profiles from multiple public microarray datasets, identifying differentially expressed mRNAs and miRNAs and constructing protein–protein interaction and miRNA–mRNA regulatory networks [17]. However, the datasets included in that analysis were generated using different microarray platforms, introducing inherent platform-specific variability that may influence gene coverage, probe design, and expression estimates. Given these limitations, a deeper understanding of the molecular mechanisms underlying VS is important for the development of targeted therapies and the identification of reliable biomarkers. Although recent studies suggest that inflammation and angiogenesis may play critical roles in tumor growth, the molecular pathogenesis of VS remains incompletely understood [7,18]. Transcriptomic profiling offers a valuable approach to address this knowledge gap. However, studies analysing the genetic profiles of patients with VS are limited and often restricted by small sample sizes.

Here, we conducted a meta-analysis using microarray datasets from VS patients in the Gene Expression Omnibus (GEO) database, incorporating analytical models that account for potential covariates. Through this approach, we aimed to comprehensively identify consistent mRNA-level alterations, drug–gene interactions, and biological functions associated with differentially expressed genes (DEGs) in VS. Moreover, we provide a comprehensive overview of the molecular pathobiology of VS by systematically examining a wide range of biological processes, from sensory processes to cell adhesion, tumour growth, immune response, and metabolic functions, using GO analyses.

## Materials and methods

### Datasets description and data preprocessing

We analysed a total of four datasets derived from GEO studies: *GSE108237, GSE141801, GSE108524 and GSE39645*. Dataset selection was primarily based on the following criteria: 1) human as the organism of study, 2) independent cohorts of patients and controls, 3) brain as a sample sources, 4) the presence of a control group consisting of healthy patients (without schwannoma), 5) the experiment type being either expression profiling by array or RNAseq, 6) Affymetrix platform to standardize analyses to the similar data preprocessing, 7) a total sample size of more than 20. Notably, a deliberate exception to the sample size threshold was made for GSE108237 (n = 14) to maximize global statistical power and capture broader microenvironmental diversity within the limited landscape of publicly available open-access datasets for this condition. To evaluate whether the meta-analysis results were disproportionately influenced by the smallest included cohort, a leave-one-out sensitivity analysis was performed by excluding GSE108237 and repeating the random-effects meta-analysis using the remaining datasets. The resulting metaLFC values, DEG counts, direction concordance, and the stability of key genes discussed in the Results were compared with those obtained from the main analysis.

The studies available in GEO were approved by the relevant regional ethics committees, as described in the corresponding publications. Covariate data, when accessible, were extracted from each dataset and incorporated into further analyses. Each meta-analysis involved two modelling approaches: a model excluding covariate adjustment (without-covariates model) and another incorporating available covariates (with-covariates model). In cases where a study lacked covariate data, the set of differentially expressed genes was the same for both models. Detailed covariate descriptions, including the specific types of variables extracted from each study (e.g., age, sex, VS type, or radiation history), are provided in S1 Table.

### Differential gene expression analysis

We analysed VS-related gene expression datasets from the GEO using the R programming language (version 4.5.1). After reading the raw data, the data quality was checked using built-in functions. For data normalization, we converted raw values into a log2 scale, utilizing the Robust Multi-Array (RMA) method, which included background correction, quantile normalization, and summarization of the probe-level data [19]. The RMA method is implemented in the *affy::expresso()* and *affy::exprs()* functions from the *affy* package. When the Affy package was not suitable for the data pre-processing and normalization, *read.celfiles()* function was used to read the raw data, and *rma()* for performing RMA from the oligo package was utilized [20,21]. Quality control was performed through visualizations, including boxplots and density plots.

Phenotype data were curated by retrieving metadata with the getGEO function from the GEOquery R package [22]. Samples were filtered to include only schwannoma and healthy control tissues, and diagnosis labels were standardized accordingly. Subsequently, a design matrix for differential expression analysis was generated using the *limma* package [23]. A linear model was fitted with diagnosis as the predictor variable, and variance estimates were moderated using empirical Bayes (eBayes) methods and standard errors were derived for each probe. Probes were filtered to include only annotated genes. Gene annotation was performed by querying the Ensembl database or using external annotation files via libraries [24]. Following batch-wise processing of probe IDs, differential expression results were combined with gene annotation information. The combined dataset was saved as an .xlsx file for further analysis.

For the covariate-adjusted analyses, differential expression modeling was performed separately within each dataset prior to meta-analysis. Available clinical covariates (including age, sex, radiation status, and VS subtype, when available) were incorporated into the limma design matrix together with diagnosis status. The resulting covariate-adjusted logFC and standard error estimates were subsequently used as inputs for the random-effects meta-analysis. Covariates were therefore incorporated at the dataset-specific differential expression stage rather than through study-level meta-regression.

### Meta-analysis

Meta-analysis of differentially expressed genes (DEGs) across multiple transcriptomic datasets was conducted using a rigorous systematic workflow. GEO datasets were obtained and processed to ensure quality control and cohort-specific differential expression analysis. Gene identifiers were standardized across datasets using gene symbol annotations, platform-specific annotation files, and Ensembl references. A random-effect meta-analysis was performed in multiple GEO datasets. Only genes represented in at least three independent studies were included in the analysis to ensure sufficient data for robust estimation. When multiple probes mapped to the same gene, probe-level values were condensed into a single gene-level estimate. For each gene, the mean log2 fold change (logFC) across all associated probes was calculated to represent the gene-level effect size, while the standard error (SE) for this estimate was taken as the maximum SE observed among the probes. This approach accounts for discordant probe signals and reduces the influence of extreme values. Random-effects meta-analyses were conducted using the Sidik–Jonkman estimator to account for between-study heterogeneity. For each gene, heterogeneity metrics (tau², I², H², and Cochran’s Q) were calculated [25]. The results, including meta-estimated log fold changes, standard errors, p-values, and heterogeneity statistics, were compiled into a consolidated dataset. Differentially expressed genes were primarily defined using an adjusted p-value (padj < 0.05) and an absolute meta-log fold change (|metaLFC| > 0.5). For descriptive purposes, the number of genes meeting nominal significance (p < 0.05) was also reported. Additionally, a more stringent threshold (|metaLFC| > 1 and padj < 0.05) was applied in downstream analyses to focus on genes with larger effect sizes. All computations were conducted in R using the metafor package (version 4.8.0) [26]. The meta-analytic procedures were performed identically for both models (without covariates and with covariates models).

### Gene Ontology (GO) enrichment analysis

To gain insight into the biological functions and pathways associated with significant DEGs, gene ontology (GO) pathway enrichment analysis was conducted using the clusterProfiler R package [27]. Gene symbols were converted to Entrez IDs using the org.Hs.e.g.,db annotation database, and enrichment analyses were carried out to identify biological processes and pathways overrepresented among the meta-significant DEGs. Functional enrichment analysis based on Gene Ontology (GO) Biological Process (BP) terms was performed to identify biological processes overrepresented among the significantly dysregulated genes identified in the meta-analysis. Gene symbols were converted to Entrez Gene identifiers using the org.Hs.e.g.,db annotation database (v3.21.0) via the mapIds() function in R. Duplicate and unmapped identifiers were removed to generate a unique and high-confidence gene list. P-values from the meta-analysis were adjusted using the Benjamini–Hochberg (BH) method to control the false discovery rate (FDR). Genes with adjusted p-values < 0.05 were considered significant and included in the enrichment analysis, while all successfully mapped genes served as the background universe. GO enrichment analysis was performed using the clusterProfiler R package (v4.16.0), employing the enrichGO() function with parameters ont = “BP,” pAdjustMethod = “BH,” pvalueCutoff = 0.05, and qvalueCutoff = 0.2. The analysis was based on the human gene annotation database (OrgDb = org.Hs.e.g.,db) and utilized Entrez IDs as the key type. Enriched GO terms were ranked according to adjusted p-values, and the top 20 most significant biological process terms were visualized as enrichment bar plots using the ggplot2 and enrichplot packages. In these plots, bar length represents −log10(adjusted p-value), while dot color indicates the number of genes associated with each enriched GO term.

### DrugBank analysis

Drug–gene interaction analysis was performed using the *dbparser* R package (v2.0.3), which enables structured parsing of DrugBank’s XML database (April 8, 2025). The full DrugBank dataset was parsed using the *parseDrugBank()* function to extract drug names, target proteins, polypeptides, general drug information and synonyms information. Only verified target interactions with “known action = yes” were retained for downstream analysis. The targets were merged with drug names, polypeptides, and synonyms using unique identifiers (drugbank_id and target_id). Gene names were standardized using the *Homo sapiens* gene reference annotation dataset to ensure consistency across datasets [28].

Significantly dysregulated genes obtained from the meta-analysis were categorized as up- or down-regulated based on the sign of meta log2 fold change (metaLFC). Gene–drug interactions were identified by merging meta-analysis results with DrugBank target gene data through gene symbol matching. The resulting drug–gene interaction pairs were filtered to retain unique, biologically verified relationships, and exported for visualization.

Network visualization of drug–gene associations was performed using the igraph (v2.2.0) and ggraph (v2.2.2) packages. To enhance interpretability, only the top 20 genes with the largest absolute effect sizes (|metaLFC|) and their interacting drugs were included in the final network plots. Nodes were color-coded according to regulation direction (up- or down-regulated genes and drug nodes), and edges represented verified interactions extracted from DrugBank.

## Results

We performed a meta-analysis with and without covariate adjustment on 21,113 genes across four schwannoma brain datasets from the GEO database. Substantial between-study heterogeneity was observed across the meta-analysed genes, with a median I² of 89.22% across the background gene set. Among genes classified as significant DEGs, 75.01% showed I² values greater than 50%, and 64.70% had significant Cochran’s Q tests (p < 0.05). This high level of heterogeneity supports the use of a random-effects framework, but also indicates that individual pooled metaLFC estimates should be interpreted with caution. To further evaluate the robustness of the findings in this context, we performed a leave-one-out sensitivity analysis excluding the smallest cohort, GSE108237. MetaLFC values were strongly correlated between the main and leave-one-out analyses across common genes (Spearman’s rho = 0.874), and 82.8% of genes retained the same direction of effect in the unadjusted model, with a similar pattern observed in the covariate-adjusted model (83.2%).

**Fig 1:**
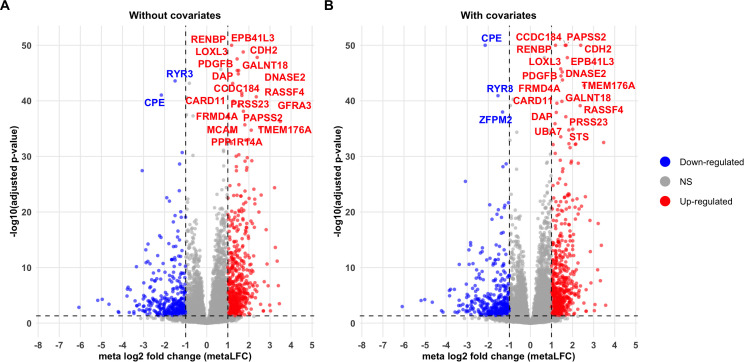
Volcano plot of meta-analysis results. This figure presents volcano plots of meta analysed top 20 genes in VS patients, divided into two panels: A) results without covariate adjustment and B) results with covariate adjustment. Each gene is displayed as an individual point, plotted according to its meta-analysed log2 fold change (metaLFC) on the x-axis and the –log10 of the adjusted p-value (padj) on the y-axis. Genes with |metaLFC| > 1 and padj < 0.05 were classified as differentially expressed, with upregulated genes shown in red and downregulated genes shown in blue. Gene labels indicate the 20 differentially expressed genes with the smallest adjusted p-values in each model.

Without including covariates, 7,354 genes reached nominal significance (p < 0.05), and 5,421 genes remained significant after adjusting for multiple comparisons (padj <0.05). Applying thresholds of |metaLFC| > 0.5 and padj < 0.05, 3,204 genes showed differential expression, including 2,132 upregulated and 1,072 downregulated genes. Raising the logFC threshold to |metaLFC| > 1 and padj < 0.05, we identified 683 upregulated and 412 downregulated genes. The overall distribution of these meta-analysed expression changes is shown in the volcano plot in Fig 1A, where gene labels indicate the 20 differentially expressed genes with the smallest adjusted p-values. Among the significant DEGs (|metaLFC| > 1 and padj <0.05), the most strongly downregulated genes based on metaLFC were *MFAP5* (−6.06), *ADH1B* (−5.17), *SLC22A3* (−4.95), *FABP4* (−4.64) and *SFRP2* (−4.2), whereas the most strongly upregulated genes based on metaLFC were*GFRA3* (3.48), *OLR1* (3.44), *GPR83* (3.39), *L1CAM* (3.34) and *ADAM23* (3.25).

Meanwhile, when covariates were included, 7,350 genes were nominally significant (p < 0.05), and 5,532 genes remained significant after multiple comparison correction (padj < 0.05). Applying a |metaLFC| > 0.5 and padj < 0.05 cutoff resulted in 3,250 differentially expressed genes, comprising 2,143 upregulated and 1,107 downregulated genes. A more stringent cutoff (|metaLFC| > 1 and padj < 0.05) reduced this set to 690 upregulated and 413 downregulated genes. The covariate-adjusted results are shown in Fig 1B, with gene labels representing the 20 significant DEGs with the smallest adjusted p-values. The strongest downregulated genes based on metaLFC were unchanged compared with the unadjusted model and showed comparable effect sizes. Among the upregulated genes, those with the highest metaLFC values were *OLR1* (3.579), *GFRA3* (3.47), *L1CAM* (3.36), *GPR83* (3.32), and *MOXD1* (3.21).

### Gene Ontology (GO) enrichment analysis

**Fig 2:**
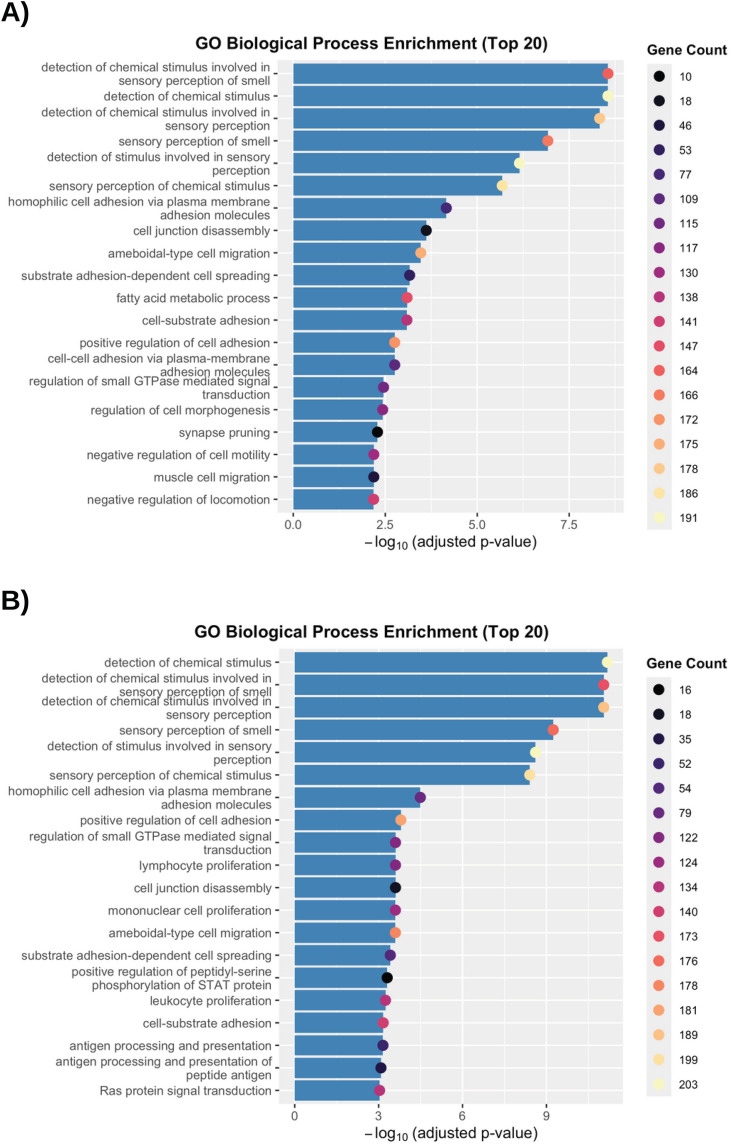
Top GO Enrichment Analysis with Gene Counts. The top 20 enriched biological processes (GO) were identified using an adjusted p-value threshold of < 0.05 (FDR-corrected) and |metaLFC| > 0.5. GO terms were ranked according to significance and visualized using bar plots, where bar length represents -log10(adjusted p-value) and dot color indicates the number of enriched genes associated with each GO term. A) the results without adjusting for covariates and B) the results with adjusting for covariates.

To investigate the functional enrichment of DEGs, we conducted a Gene Ontology (GO) enrichment analysis on significant genes identified from two meta-analysis models, applying a significance threshold of |metaLFC| > 0.5 and padj < 0.05. We extracted the top 20 biological processes (BP) based on the lowest adjusted p-values (false discovery rate, FDR). The GO analysis results are presented in Fig 2. In the model without covariates, top biological processes were predominantly associated with sensory perception and cell adhesion dynamics, including cell junction disassembly, amoeboidal-type migration, and substrate adhesion-dependent cell spreading (Fig 2A). We used bar plots for each graph, with dot colour indicating enriched gene count per pathway. A total of 115 significant GO term enrichments were identified in the model without covariates, and are provided in S2 Table.

In the with covariates model, among the top enriched terms, processes associated with the detection of chemical stimulus and sensory perception, including the detection of sensory stimulus involved in smell, showed the highest statistical significance. In addition, several signalling-related pathways, such as Ras protein signal transduction and regulation of small GTPase-mediated signal transduction, were significantly enriched, indicating the involvement of intracellular signalling mechanisms. Also, immune-related processes, including antigen processing and presentation and lymphocyte proliferation were prominently represented, suggesting a potential role of immune regulation within the analysed gene set. The top 20 significantly enriched biological processes are shown in Fig 2B, while the complete list of 170 enriched GO terms is provided in S3 Table.

### DrugBank analysis

**Fig 3:**
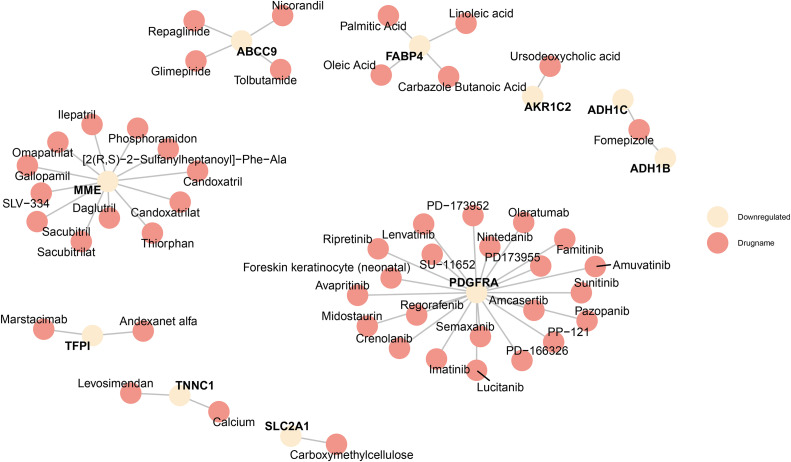
Drug-gene Interaction Network for the Top 10 Downregulated Genes. Networks were constructed using experimentally supported drug-target interactions from DrugBank, focusing on significantly differentially expressed genes (|metaLFC| > 1 and padj < 0.05).

**Fig 4:**
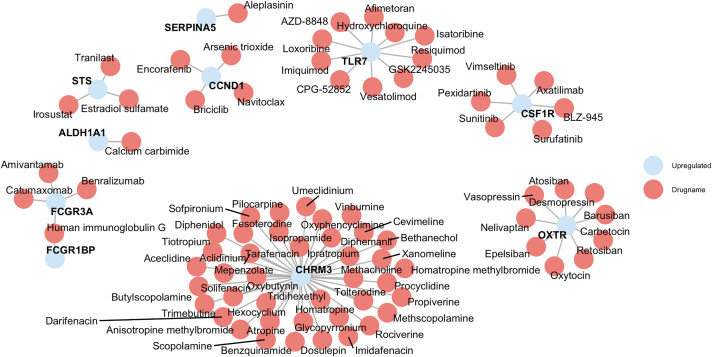
Drug-gene Interaction Network for the Top 10 Upregulated Genes. Networks were constructed using experimentally supported drug-target interactions from DrugBank, focusing on significantly differentially expressed genes (|metaLFC| > 1 and padj < 0.05).

DrugBank analysis revealed pharmacological associations between the DEGs and several known drugs, and possible pharmacological relationships. The *ADH1B* gene was downregulated, and an interaction between this gene and fomepizole was identified. Fomepizole was also associated with *ADH1C*, another downregulated alcohol dehydrogenase gene. Fomepizole is known as a competitive inhibitor of alcohol dehydrogenase enzymes. Also, the gene *FCGR3A*, which was upregulated, was found to interact with human immunoglobulin G (IgG). The *FCGR3A* gene may affect the binding capacity of IgG. A total of 154 genes displaying significant drug–gene interactions were identified using the stringent threshold of |metaLFC| > 1 and padj < 0.05. Among these, the top 10 downregulated and top 10 upregulated genes, ranked by metaLFC values, are presented in Figs 3 and 4, respectively. Several genes were found to interact with multiple drugs, resulting in a cumulative total of 1009 drug–gene interactions. The full list of identified interactions is provided in the S4 Table.

## Discussion

In this study, we conducted the first transcriptome-wide meta-analysis of vestibular schwannoma (VS), integrating multiple independent Affymetrix-based microarray cohorts to derive a statistically robust and platform-homogeneous assessment of genome-wide transcriptional alterations. Using a random-effects meta-analytic framework, we identified more than 3,200 differentially expressed genes (DEGs), based on a cutoff of |metaLFC| > 0.5 and padj < 0.05, with a high degree of concordance between the covariate-adjusted and unadjusted models. Application of a more stringent threshold (|metaLFC| > 1, padj < 0.05) yielded approximately 1,100 DEGs in both analytical conditions, underscoring the stability and reproducibility of the observed transcriptional signals. Functionally, these DEGs were enriched for several broad biological themes, including pronounced downregulation of extracellular matrix and stromal components (e.g., *MFAP5, FABP4, DCN*), alterations in sensory- and synapse-related genes implicated in auditory function (such as *SLC22A3* and *LGI1*), and increased expression of immune- and inflammation-associated pathways (including *TREM2*, *CCL3*, and *CCL4*) alongside proliferative regulators (notably *CCND1* and *RAB31*). Notably, many of the most strongly dysregulated genes identified in this meta-analysis have not previously been evaluated in VS, highlighting the added value of a genome-wide, discovery-oriented approach. Collectively, these quantitative findings are consistent with a multifaceted transcriptional landscape in VS that may involve contributions from tumor-intrinsic and microenvironment-associated processes, including immune-related signaling and tumor-associated growth pathways; however, further validation using complementary and higher-resolution transcriptomic approaches (e.g., single-cell, spatial, or integrative multi-cohort analyses) will be required to clarify the underlying cellular and biological context. These findings may also help prioritize candidate genes and pathways for future biomarker development and therapeutic investigation. To contextualize our findings, we compared our DEGs with previously published VS studies, including bulk microarray analyses and recent RNA-seq–derived signature studies [14–16]. For upregulated genes, several were consistently identified across datasets, most notably *NRG1*, along with *SPP1* and *CDH2*, supporting prior VS transcriptomic findings. Consistent with previous studies [14,15], *SLC22A3* and *PRRX1* were significantly downregulated in our study. Additional genes, including *FABP4*, *MFAP5*, and *SFRP2* [15], as well as *TNNC1*, *EBF1*, and *EBF2* [14], were shared between two studies, indicating a combination of conserved and context-dependent transcriptional changes. *GFRA3*, *GPR83*, and *MOXD1* were among the most strongly dysregulated genes in our dataset and have not been prominently reported in previous VS transcriptomic studies, suggesting that they may represent additional candidate genes requiring further validation.

Partial concordance with the 24-gene communication signature proposed by Sung et al. was observed, primarily driven by *SPP1* and members of the CCL chemokine family, including *CCL3* and *CCL4*, indicating shared enrichment of immune and macrophage-associated pathways [16]. In addition, we assessed the overlap between the 24-component Schwannoma signature reported by Sung et al. and the results of our meta-analysis. Since Sung et al. define the schwannoma signature as a 24-gene set in their article and corresponding Supplementary Table 4, we used the 24-component version for this comparison. Nineteen of the 24 signature components (79.2%) were represented by at least one significantly differentially expressed gene in our study. This component-level comparison identified partial recovery of the corresponding gene families or signaling groups, whereas gene-level overlap and directionality are provided separately in S5 Table. Furthermore, genes reported in recent VS single-cell studies, including *SPP1* and *NRG1*, were also identified as upregulated in our dataset [29,30]. In addition, pathways related to macrophage infiltration and cell–cell communication described in these studies are supported by the differential expression of genes such as CCL3, CCL4, and TREM2 in our analysis [31]. Together, these findings suggest that key microenvironmental and intercellular signaling features observed at the single-cell level are partially reflected in our meta-analysis data. The limited gene-level overlap observed across studies is methodologically expected and should be interpreted in the context of differences in sequencing platforms, statistical thresholds, and growth-stratification strategies, all of which can influence DEG detection.

One of the most prominent and consistent transcriptional features identified in this meta-analysis was the marked downregulation of extracellular matrix (ECM)– and stroma-associated genes in VS. The strongest effect size across the entire dataset was observed for *MFAP5* (metaLFC = −6.06), an ECM-associated protein implicated in microfibril organization and stromal structure [32]. In addition to *MFAP5*, several genes involved in extracellular matrix integrity and stromal organization were among the most significantly downregulated transcripts, including *FABP4* (metaLFC = −4.64), *DCN* (metaLFC = −3.67), *OMD* (metaLFC = −4.17), and *SFRP2* (metaLFC = −4.20). The magnitude of suppression across these genes points toward a large-scale alteration of the extracellular architecture. While *MFAP5* expression has been reported to be elevated in stromal compartments of certain malignant tumors, particularly within cancer-associated fibroblasts, its strong downregulation in VS suggests a context-dependent regulatory pattern that may reflect tumor-type–specific stromal remodeling [32–35]. Nevertheless, it remains to be elucidated whether these changes primarily occur within the neoplastic Schwann cells or reflect a relative depletion or phenotypic shift of stromal fibroblast populations within the tumor bulk. To our knowledge, *MFAP5* and several of the accompanying ECM-related genes identified here have not previously been evaluated in VS at the transcriptome-wide level. Collectively, these findings suggest that disruption of extracellular matrix composition and stromal organization is a prominent transcriptional feature of VS.

Beyond extracellular matrix remodeling, our meta-analysis revealed consistent downregulation of genes implicated in sensory support and synaptic function, which may reflect gene expression alterations relevant to auditory physiology in VS. *SLC22A3* was among the most strongly downregulated genes (metaLFC = −4.95). This gene encodes an organic cation transporter expressed in supporting cells across sensory epithelia and is involved in monoaminergic signaling and glial homeostatic functions [36]. Experimental evidence indicates that *SLC22A3*-mediated transport influences serotonin availability, epigenetic regulation, and inhibitory neurotransmission, processes relevant to sensory signal modulation [37]. In line with its proposed regulatory role, *SLC22A3* downregulation has been reported across multiple cancer types, consistent with a hypothesized tumor suppressor function [38,39]. In addition to *SLC22A3*, *LGI1*—a gene highly expressed in neuronal tissues and involved in synaptic stability—was significantly downregulated in VS (metaLFC = −2.55). Reduced *LGI1* expression has previously been associated with glial tumors and neurological disorders featuring auditory manifestations [40,41]. These findings are consistent with gene expression changes affecting sensory and synaptic pathways. However, the bulk nature of the data does not allow resolution of cell-type–specific contributions or causal relationships. Within this context, the concurrent suppression of *SLC22A3* and *LGI1* may represent a molecular correlate of auditory dysfunction in VS that is not solely explained by tumor mass effects and warrants further investigation using higher-resolution transcriptomic approaches. A further prominent feature of the VS transcriptome identified in this meta-analysis was the upregulation of genes associated with immune and inflammatory processes, suggesting transcriptional enrichment of these pathways in VS. Among these, *L1CAM* was significantly upregulated (metaLFC = 3.34). Although classically involved in neural cell adhesion, aberrant *L1CAM* expression has been widely reported in cancer and linked to inflammatory signaling, including activation of NF-κB–dependent pathways [42]. Consistent with prior transcriptomic studies reporting inflammatory pathway enrichment in VS, the observed *L1CAM* upregulation may reflect inflammation-associated transcriptional changes within the tumor microenvironment [43]. In addition, *TREM2* was significantly upregulated (metaLFC = 2.58). *TREM2* expression has been shown to correlate with immune cell infiltration in VS [44]. In line with this association, the chemokines *CCL3* and *CCL4* were also markedly upregulated in our analysis (metaLFC = 3.02 and 2.98, respectively). These CC chemokines are key mediators of immune cell recruitment, including monocytes, macrophages, dendritic cells, and T cells [45]. Together, the coordinated upregulation of *L1CAM, TREM2, CCL3*, and *CCL4* may suggest immune-associated gene expression in VS. These immune-related gene expression changes may, in part, reflect contributions from non-neoplastic components of the tumor microenvironment.

Genes associated with cell cycle regulation and tumor growth pathways were also consistently upregulated in VS. Among these, *CCND1* was significantly increased in the meta-analysis (metaLFC = 2.16). *CCND1* encodes cyclin D1, a key regulator of G1–S phase cell cycle progression, and its dysregulation has previously been associated with increased risk and growth of intracranial tumors, including acoustic neuroma [46]. Elevated *CCND1* expression has been shown to promote cellular proliferation and tumor expansion across multiple tumor types, suggesting that its upregulation in VS may be consistent with a transcriptional state supporting sustained, non-malignant growth tendencies [47,48]. In addition, *RAB31* was among the most strongly upregulated genes (metaLFC = 2.91). *RAB31* is a member of the Ras-related small GTPase family and has been associated with pathways related to tumor development, survival signaling, and resistance to apoptosis in several cancers, including glioma [49]. Functional studies in other tumor systems have linked *RAB31* overexpression to enhanced proliferative capacity and reduced cell death [49]. To date, *RAB31* has not been evaluated in the context of VS, and its upregulation may reflect shared features of growth- and survival related transcriptional patterns. These associations resemble transcriptional patterns linked to proliferation and survival in other tumors, although the current meta-analysis of bulk transcriptomic data cannot determine whether these signals originate from tumor cells, stromal components, or infiltrating immune cells.

Developmental and neural crest–associated transcriptional patterns were evident in VS. *MOXD1* was significantly upregulated (metaLFC = 3.21) and is known to be enriched in neural crest–derived Schwann cell precursors [50]. While *MOXD1* expression has been associated with aggressive tumor behavior in glioblastoma, its loss in neuroblastoma correlates with advanced disease, highlighting a context-dependent role across neural crest–related tumors [51,52]. In the context of VS, a benign Schwann cell–derived neoplasm, *MOXD1* upregulation may reflect developmental gene expression features rather than malignant progression. The concurrent upregulation of *MOXD1* alongside proliferative, sensory, and immune-associated gene expression changes may indicate broader developmental influences within the VS transcriptome, potentially affecting interactions within the tumor microenvironment [51].

Our meta-analysis identified a substantial network of 1,009 drug–gene interactions, providing a pharmacological landscape that partially overlaps with the VS transcriptome. DrugBank analysis revealed that several DEGs intersect with previously characterized pharmacological targets, providing a descriptive, hypothesis-generating framework for candidate prioritization. Several factors constrain direct clinical inference: the blood–brain barrier limits central nervous system bioavailability for many compounds, druggability in the VS context remains unestablished for the majority of identified targets, and tumor specificity and systemic toxicity profiles have not been evaluated. Nonetheless, such mappings may support the prioritization of candidate targets for future mechanistic and preclinical studies.

Various clinical and biological covariates may influence gene expression patterns in VS. In a multiplatform molecular analysis, no significant differences in age or sex were detected between VS subgroups [53]. Similarly, a review on VS tumor growth reported that sex is not a determining factor, while age has been suggested as a potential determinant in only a limited number of studies [54]. The same review also indicated that tumors with cystic components may represent a significant risk factor for tumor growth. Given the potential influence of such variables, covariates including age, sex, radiation status, VS type, and disease state were considered in our analyses when available. To address potential biases arising from the inconsistent availability of clinical metadata in public repositories, we adopted a dual-modeling approach with and without covariate adjustment. As shown in S1 Table, covariate availability varied across studies: while datasets such as GSE141801 provided detailed information on radiation history and VS subtypes, others (e.g., GSE39645) lacked clinical metadata. Although missing covariates may theoretically introduce residual confounding, the high consistency between adjusted and unadjusted models suggests that the identified gene expression signatures are only weakly influenced by age, sex, and disease state.

A key strength of this study lies in its comprehensive meta-analytic design, which integrated multiple independent Affymetrix-based microarray datasets to generate robust, cross-cohort transcriptional signatures while minimizing technical heterogeneity. The inclusion of both covariate-adjusted and non-adjusted models demonstrated high concordance of differential expression, reinforcing the reproducibility of the observed alterations. The genome-wide, hypothesis-free approach enabled identification of convergent biological themes—including extracellular matrix remodeling, sensory- and synapse-related pathways, immune activation, and proliferative signaling—rather than isolated gene-specific effects. Notably, drug–gene interaction mapping uncovered a substantial number of pharmacological associations, suggesting that parts of the VS transcriptomic landscape intersect with existing drug targets. Nonetheless, several limitations should be acknowledged. The number of publicly available VS datasets remains limited, reflecting the challenges of obtaining surgical tissue and restricting opportunities for large-scale, standardized transcriptomic studies. In addition, bulk transcriptome profiling precludes direct resolution of cell-type–specific contributions and spatial heterogeneity within the tumor microenvironment. Despite these constraints, the integrated transcriptional framework provided here delineates a multifaceted molecular landscape of VS, highlights previously uncharacterized genes and pathways, and establishes a foundation for future functional studies and potential translational applications, including biomarker discovery and mechanistic investigation of tumor growth and auditory dysfunction.

In conclusion, this meta-analysis defines a multifaceted transcriptional landscape in VS, yet several key questions remain. The cellular sources of immune and stromal alterations, the functional consequences of sensory- and synapse-related gene dysregulation, and the mechanistic role of previously uncharacterized genes such as *MFAP5*, *RAB31*, and *MOXD1* remain incompletely understood. Future studies integrating single-cell and spatial transcriptomics, longitudinal growth analyses, and functional validation will be essential to clarify the molecular mechanisms driving tumor progression, sensory deficits, and microenvironmental interactions, ultimately informing biomarker development and therapeutic strategies in VS.

## Supporting information

S1 Table GEO Dataset Metadata (Covariates and Demographics). (XLSX)

S2 Table Gene Ontology (GO) term enrichment results from the model without covariates. (XLSX)

S3 Table Gene Ontology (GO) term enrichment results from the model with covariates. (XLSX)

S4 Table Complete list of 1009 drug–gene interactions identified through DrugBank analysis. (XLSX)

S5 Table Comparison between the 23-component Schwannoma signature reported by Sung et al. (2024) and the results of our meta-analysis. (DOCX)

## Abbreviations

BP: Biological Process
DEGs: Differentially Expressed Genes
FDR: False Discovery Rate
GEO: Gene Expression Omnibus
GO: Gene Ontology
logFC: log2 fold change
RMA: Robust Multi-Array
SE: Standard Error
SJ: Sidik–Jonkman estimator
VS: Vestibular Schwannoma
