# Supplementary material for: Transcriptomic meta-analysis identifies dysregulated pathways and potential therapeutic targets in Vestibular Schwannoma
Source: PLoS One. 2026 Jul 10;21(7):e0353343. doi: 10.1371/journal.pone.0353343 (PMC13353982; doi:10.1371/journal.pone.0353343)
Supplement: S5 Table — (DOCX) [file pone.0353343.s005.docx]

**S5 Table.** Comparison between the 23-component Schwannoma signature reported by Sung et al. (2024) and the results of our meta-analysis. A signature component was considered represented if at least one significantly differentially expressed gene (adjusted p < 0.05) belonging to the corresponding gene family or signaling group was identified. Values in parentheses indicate meta-estimated log2 fold changes.

| **Gene family from Sung et al. (2024)** | **Genes from our study** |
| --- | --- |
| CXCL | *CXCL10* (1.06), *CXCL16* (1.58), *CXCL8* (-1.21) |
| GALECTIN | *LGALS3BP* (1.74), *LGALS9* (1.77), *LGALS9B* (1.34), *LGALS9C* (1.33) |
| COMPLEMENT | *CFH* (-3.46), *C1QA* (1.75), *C1QB* (1.81), *C1QC* (1.95), *C4B* (-1.67), *C3* (1.69), *C6* (-1.2), *C7* (-2.4), *C3AR1* (1.93), *CR1* (-1.73), *ITGAM* (1.36), *ITGB2* (1.57) |
| MK (MDK) | absent |
| VISFATIN (NAMPT) | *NAMPT* (-0.72) |
| PSAP | *PSAP* (0.72) |
| VEGF | *VEGFB* (1.47) |
| GRN | *GRN* (1.37) |
| PTN | *PTN* (0.65) |
| SPP1 | *SPP1* (2.1) |
| WNT | absent |
| ANNEXIN | *ANXA3* (1.15) |
| CCL | *CCL14* (-1.24), *CCL15* (-1.07), *CCL15-CCL14* (-1.19), *CCL3* (3.02), *CCL3L1* (2.68), *CCL3L3* (2.68), *CCL4* (2.99), *CCL4L1* (2.56), *CCL4L2* (2.31) |
| PROS | absent |
| GAS | *GAS2* (-1.11) |
| PDGF | *PDGFA* (1.26), *PDGFB* (1.45), *PDGFD* (1.85) |
| ANGPT | *ANGPT1* (-2.35) |
| PAR | *PARD3* (-1.12), *PARM1* (-2.03), *PARP12* (0.66), *PARP3* (0.62), *PARP9* (0.76), *PARVG* (1.15) |
| FGF | *FGF2* (1.26), *FGF10* (-1.22), *FGF20* (1.12) |
| EDN | *EDN1* (-0.91) |
| IGF | *IGFBP4* (1.72), *IGFBP5* (-1.25), *IGFBP6* (-1.11), *IGFBP2* (1.89) |
| EGF | absent |
| CS3C | absent |
| BMP | *BMP7* (1.35), *BMP6* (1.76), *BMP8B* (1.17), *BMP5* (-1.44), *BMP3* (1.89) |
